# Enhanced Discrimination of Coronary Artery Disease Severity by Circulating Phoenixin-14: Evidence from a Clinical Laboratory Study

**DOI:** 10.3390/ijms27135719

**Published:** 2026-06-24

**Authors:** İsmail Polat, Bekir Dagdeviren, Mehdi Karasu, Ömer Bedir, Suna Aydin, Elif Emre, Musa Sari, Özlem Seçen, Çetin Mirzaoglu, Suleyman Aydin

**Affiliations:** 1 Department of Cardiology, Fethi Sekin City Hospital, 23119 Elazig, Turkey; drismailpolat49@gmail.com (İ.P.); mehdikarasu@yahoo.com (M.K.);; 2 Department of Physiotherapy and Rehabilitation, Faculty of Health Sciences, Firat University, 23119 Elazig, Turkey; bdagdeviren@firat.edu.tr; 3 Department of Cardiology, Adana City Training and Research Hospital, 01000 Adana, Turkey; dromerbedir@gmail.com; 4 Department of Cardiovascular Surgery, Fethi Sekin City Hospital, 23119 Elazig, Turkey; cerrah52@hotmail.com; 5 Department of Anatomy, Faculty of Medicine, Fırat University, 23119 Elazig, Turkey; eemre@firat.edu.tr; 6 Department of Biology (Molecular Biology), Faculty of Science, Sivas Cumhuriyet University, 58000 Sivas, Turkey; 7 Department of Biochemistry, Faculty of Medicine, Fırat University, 23119 Elazig, Turkey

**Keywords:** coronary artery disease, biomarkers, Phoenixin-14, Syntenin-1, Alamandine, Cerebellin-1, coronary stenosis, ROC analysis, stable angina pectoris, diagnostic accuracy

## Abstract

Early identification of anatomically significant coronary artery disease (CAD) remains a major clinical challenge despite advances in cardiovascular diagnostics. Novel circulating biomarkers may improve risk stratification and diagnostic discrimination beyond conventional parameters. We investigated the diagnostic utility of four emerging biomarkers—Phoenixin-14, Syntenin-1, Alamandine, and Cerebellin-1—for the assessment of CAD severity. In this prospective observational study, 90 participants undergoing coronary angiography were categorized into three groups: severe CAD (≥70% stenosis; *n* = 30), non-obstructive/non-critical CAD (<70% stenosis; *n* = 30), and angiographically normal controls (*n* = 30). Patients with acute coronary syndrome, diabetes mellitus, prior coronary revascularization, cardiomyopathy, or significant systemic disease were excluded. Circulating biomarker concentrations were quantified using the enzyme-linked immunosorbent assay. Comparative analyses, correlation testing, and receiver operating characteristic (ROC) analyses were performed to evaluate discriminatory performance. Circulating Phoenixin-14 concentrations progressively declined across the control, non-critical CAD, and severe CAD groups [40.1 (29.0–49.7) vs. 24.4 (18.5–30.1) vs. 16.7 (13.4–19.0) pg/mL, respectively; *p* < 0.001]. Phoenixin-14 demonstrated outstanding discrimination for severe CAD, achieving an area under the ROC curve (AUC) of 0.969 (95% CI, 0.888–0.997), with 86.7% sensitivity and 96.7% specificity at a threshold of ≤20.2 pg/mL. Diagnostic performance was substantially lower for Syntenin-1 (AUC, 0.795), Alamandine (AUC, 0.661), and Cerebellin-1 (AUC, 0.597). Phoenixin-14 also showed robust discrimination for non-critical CAD (AUC, 0.832). Biomarker concentrations exhibited correlations with metabolic indices while remaining largely independent of traditional cardiovascular risk factors. Among the evaluated novel circulating biomarkers, Phoenixin-14 demonstrated superior diagnostic performance for both obstructive and non-obstructive CAD, markedly outperforming Syntenin-1, Alamandine, and Cerebellin-1. These findings identify Phoenixin-14 as a promising candidate biomarker for CAD severity assessment and clinical risk stratification. Larger multicenter studies are warranted to validate these exploratory findings and determine their incremental value in contemporary cardiovascular practice.

## 1. Introduction

Cardiovascular disease (CVD) remains the leading cause of mortality worldwide, with coronary artery disease (CAD) representing its most prevalent manifestation and a major contributor to morbidity, heart failure, and premature death [1]. Despite substantial advances in interventional cardiology, imaging modalities, and guideline-directed medical therapies, accurately identifying patients with clinically significant coronary stenosis remains a major challenge in contemporary cardiovascular practice [2]. Invasive coronary angiography continues to represent the diagnostic gold standard; however, a considerable proportion of patients undergoing invasive evaluation are ultimately found to have non-obstructive disease or angiographically normal coronary arteries. Consequently, there is a growing need for novel circulating biomarkers capable of improving risk stratification, refining patient selection for invasive procedures, and identifying individuals at increased risk of progressive atherosclerotic disease.

The development of cardiovascular biomarkers has profoundly transformed diagnostic and prognostic strategies over the past two decades [3]. The introduction of cardiac troponins and subsequent high-sensitivity assays revolutionized the diagnosis and management of acute coronary syndromes by enabling the detection of minimal myocardial injury [4,5]. Nevertheless, conventional biomarkers predominantly reflect myocardial necrosis or hemodynamic stress rather than the underlying biological processes driving coronary atherosclerosis. CAD is a complex and multifactorial disease characterized by chronic vascular inflammation, endothelial dysfunction, oxidative stress, neurohormonal activation, metabolic dysregulation, and maladaptive cellular signaling pathways [6,7]. Therefore, biomarkers reflecting these upstream pathophysiological mechanisms may provide incremental diagnostic and prognostic value beyond currently established markers.

In parallel with advances in genomics and precision medicine, genome-wide association studies and multi-omics approaches have identified numerous loci and molecular pathways associated with cardiovascular risk. However, the translation of these discoveries into clinically applicable tools remains limited, particularly for stable CAD and disease severity assessment. Current risk prediction models still rely heavily on traditional cardiovascular risk factors, which incompletely capture the biological heterogeneity of coronary atherosclerosis. These limitations underscore the need for novel circulating biomarkers that more accurately reflect vascular injury, inflammatory signaling, endothelial impairment, and neurohormonal regulation.

Among the emerging candidates, Phoenixin-14 has recently attracted attention because of its pleiotropic regulatory effects on metabolic homeostasis, inflammatory pathways, oxidative stress, and cardiovascular function. Phoenixin is a conserved neuropeptide derived from the small integral membrane protein 20 (SMIM20) precursor and is expressed in both central and peripheral tissues, including the heart and vascular endothelium. Experimental studies suggest that Phoenixin exerts anti-inflammatory and cytoprotective effects through the modulation of mitochondrial function, endothelial signaling, and cellular survival pathways. Reduced circulating Phoenixin-14 concentrations have been associated with metabolic disorders, obesity, hypertension, and inflammatory states, supporting its potential role in cardiovascular pathophysiology [8,9].

Syntenin-1, an intracellular PDZ-domain-containing adaptor protein involved in membrane trafficking and signal transduction, has also emerged as a potential mediator of vascular remodeling and inflammatory activation. Syntenin-1 participates in exosome biogenesis, endothelial communication, and cell adhesion signaling, processes that are increasingly recognized as contributors to atherosclerotic plaque development and progression. Altered Syntenin-1 expression has been implicated in inflammatory and proliferative vascular responses, although its clinical significance in CAD remains incompletely understood [10,11].

Alamandine, a recently identified peptide of the protective arm of the renin–angiotensin system (RAS), exerts vasodilatory, anti-inflammatory, antifibrotic, and antioxidative effects predominantly through activation of the Mas-related G protein-coupled receptor D (MrgD) [12,13]. Experimental studies have demonstrated that Alamandine counteracts the detrimental cardiovascular effects of angiotensin II by improving endothelial function, reducing oxidative stress, attenuating vascular inflammation, and suppressing adverse vascular remodeling. In animal and cellular models, Alamandine signaling has been associated with enhanced nitric oxide bioavailability, inhibition of pro-inflammatory cytokine production, and protection against endothelial injury, supporting its role as a potentially protective mediator in cardiovascular disease and atherosclerosis [14]. Cerebellin-1, a member of the cerebellin precursor protein family traditionally associated with synaptic organization and neuroendocrine signaling, has recently been implicated in cardiometabolic regulation and inflammatory signaling pathways. Emerging evidence suggests that Cerebellin-1 may influence autonomic regulation, energy metabolism, and vascular responses, although its role in coronary atherosclerosis remains largely unexplored [15,16].

Collectively, these novel biomarkers represent biologically distinct yet potentially complementary pathways involved in the initiation and progression of coronary artery disease. Their integration into multimarker strategies may improve the identification of patients with clinically significant coronary stenosis and provide mechanistic insights beyond conventional biomarkers alone [17].

Therefore, the present study aimed to evaluate the diagnostic performance of Phoenixin-14, Syntenin-1, Alamandine, and Cerebellin-1 in differentiating critical from non-critical coronary artery stenosis and to investigate their associations with established cardiovascular risk factors and clinical characteristics in patients undergoing coronary angiography.

## 2. Results

### 2.1. Baseline Characteristics and Clinical Parameters

A total of 90 participants were enrolled in this study, comprising 30 patients with severe coronary stenosis, 30 patients with non-critical coronary artery disease, and 30 healthy controls matched for age and cardiovascular risk factors. The study population demonstrated balanced demographic characteristics, with no statistically significant differences in gender distribution across groups (*p* = 0.366) or smoking history (*p* = 0.574).

Hypertension prevalence differed significantly between groups (*p* = 0.013), with higher rates observed in both coronary disease cohorts compared to healthy controls (severe CAD: 60.0%, non-critical CAD: 70.0%, controls: 33.3%). Body weight varied significantly across groups (*p* = 0.037), primarily driven by elevated weight in the non-critical CAD group (81.7 ± 10.8 kg) compared to both the severe CAD (74.7 ± 11.9 kg) and control groups (74.9 ± 12.7 kg). Body mass index demonstrated significant inter-group differences (*p* = 0.003), with the severe CAD group exhibiting lower BMI values (26.0 ± 3.4 kg/m^2^) compared to the non-critical CAD group (29.6 ± 3.9 kg/m^2^).

Traditional cardiovascular risk markers demonstrated patterns consistent with increasing coronary artery disease burden. Systolic blood pressure differed significantly across study groups (*p* = 0.003), with patients in both the severe CAD and non-critical CAD groups exhibiting higher systolic values compared with the controls (136.3 ± 18.3 mmHg and 134.1 ± 14.4 mmHg vs. 122.1 ± 16.6 mmHg, respectively). In addition, diastolic blood pressure was significantly elevated in the non-critical CAD group relative to the control group (81.4 ± 10.6 vs. 74.1 ± 10.4 mmHg, *p* = 0.047). Renal function indices also differed significantly between groups. Patients with angiographically documented coronary artery disease demonstrated higher serum creatinine concentrations and lower estimated glomerular filtration rates compared with healthy controls, suggesting an association between impaired renal function and atherosclerotic disease burden (*p* < 0.001 for glomerular filtration rate). Lipid profile analysis revealed a progressive reduction in high-density lipoprotein (HDL) cholesterol levels with increasing disease severity. HDL cholesterol concentrations were lowest in the severe CAD group (39.4 ± 8.2 mg/dL), significantly lower than those observed in both the non-critical CAD group (45.4 ± 9.1 mg/dL) and the control group (49.2 ± 10.0 mg/dL; *p* < 0.001). These findings are consistent with the well-established inverse relationship between HDL cholesterol and the extent of coronary atherosclerosis (Table 1).

Medication use did not differ significantly across groups for any drug class (all *p* > 0.10; Table 2) and was balanced between the severe and non-critical CAD subgroups.

### 2.2. Circulating Biomarker Analysis

**Figure 1:**
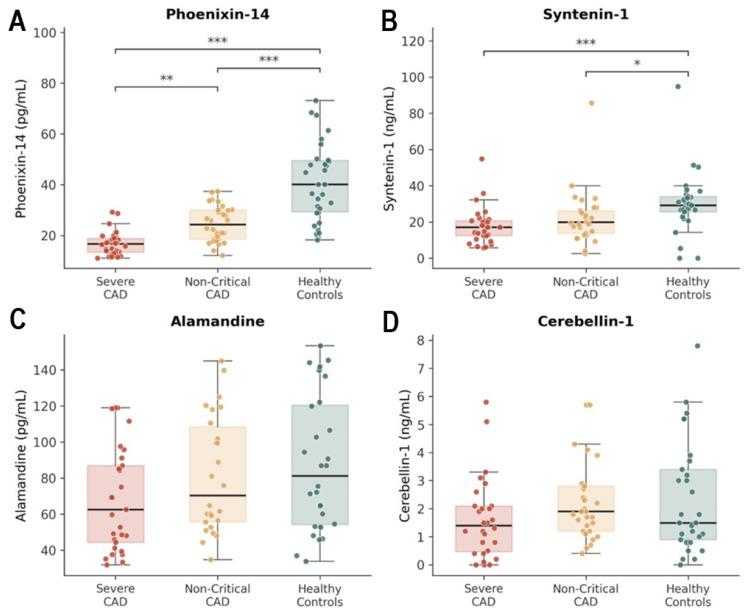
(**A**). Circulating Phoenixin-14 concentrations across study groups. (**B**). Circulating biomarker Systenin-1 concentrations across study groups. (**C**). Circulating Alamandine concentrations across study groups. (**D**). Circulating Cerebellin-1 concentrations across study groups. Box plots show the median and interquartile range; whiskers extend to 1.5 × IQR; overlaid points represent individual participants. * *p* < 0.05, ** *p* < 0.01, *** *p* < 0.001 (Dunn’s test with Bonferroni adjustment). CAD: coronary artery disease.

The four investigated circulating biomarkers exhibited heterogeneous expression patterns across the spectrum of coronary artery disease severity, with Phoenixin-14 demonstrating the most robust and clinically relevant discriminatory profile. Phoenixin-14 concentrations showed a clear stepwise reduction from the controls to non-critical CAD and further to severe coronary stenosis [40.1 (29.0–49.7) vs. 24.4 (18.5–30.1) vs. 16.7 (13.4–19.0) pg/mL, respectively; *p* < 0.001]. This graded decline remained significant across all intergroup comparisons, indicating a strong and consistent association with increasing atherosclerotic burden throughout the coronary disease continuum (Figure 1A).

Syntenin-1 levels demonstrated a statistically significant inverse pattern, with higher concentrations observed in the control group compared with both CAD subgroups (30.1 ± 17.2 ng/mL vs. 22.4 ± 15.0 ng/mL in non-critical CAD and 17.9 ± 10.2 ng/mL in severe CAD; *p* = 0.007). Although this pattern suggests a potential relationship with coronary pathology, the biological directionality and mechanistic interpretation warrant further investigation (Figure 1B).

Alamandine levels showed a decreasing trend across groups, with the lowest concentrations observed in patients with severe CAD [62.5 (44.4–87.0) pg/mL], followed by non-critical CAD [70.4 (55.6–110.6) pg/mL], and the highest values in the controls [81.3 (53.9–121.1) pg/mL]. However, these differences did not reach statistical significance (*p* = 0.075), indicating limited discriminatory performance in the present cohort (Figure 1C).

Cerebellin-1 concentrations were comparable across all study groups, with substantial overlap in distribution and no statistically significant intergroup differences [severe CAD: 1.4 (0.5–2.1), non-critical CAD: 1.9 (1.2–2.8), controls: 1.5 (0.9–3.4) ng/mL; *p* = 0.193]. These findings suggest that Cerebellin-1 does not meaningfully discriminate between coronary disease phenotypes in this population (Figure 1D).

After Bonferroni correction across the four biomarkers, omnibus group differences remained significant for Phoenixin-14 (corrected *p* < 0.001) and Syntenin-1 (corrected *p* < 0.001), whereas Alamandine and Cerebellin-1 were non-significant. For Phoenixin-14, all three pairwise comparisons remained significant after Bonferroni adjustment (severe vs. control, *p* < 0.001; non-critical vs. control, *p* < 0.001; severe vs. non-critical, *p* = 0.002).

### 2.3. Correlation Analysis and Biomarker Interactions

After Benjamini–Hochberg correction across all 82 pairwise correlations, only the positive correlation between Phoenixin-14 and Alamandine remained significant (*r* = 0.725; FDR-adjusted *p* < 0.001). The remaining nominally significant associations—Phoenixin-14 with body mass index (*r* = 0.382) and weight (*r* = 0.361), Alamandine with glucose (*r* = 0.308), and Cerebellin-1 with triglycerides (*r* = 0.262)—did not retain significance after correction for multiple comparisons (Table 3). A strong positive correlation was observed between the Alamandine and Phoenixin-14 levels (*r* = 0.725, *p* < 0.001), suggesting a closely related or potentially interdependent regulatory behavior within cardioprotective pathways. Alamandine also showed a moderate but significant positive correlation with the plasma glucose levels (*r* = 0.308, *p* = 0.022), indicating a possible link between this peptide and metabolic homeostasis. Phoenixin-14 was positively correlated with anthropometric indices, including body weight (*r* = 0.361, *p* = 0.005) and body mass index (*r* = 0.382, *p* = 0.003), supporting a potential association between this biomarker and overall metabolic status, which may indirectly contribute to cardiovascular risk modulation. Cerebellin-1 demonstrated a weak but statistically significant positive correlation with triglyceride levels (*r* = 0.262, *p* = 0.049); however, given its limited discriminatory capacity in group comparisons, the clinical relevance of this association remains uncertain.

In contrast, traditional cardiovascular risk factors including age, blood pressure parameters, and established lipid profile components showed generally weak or non-significant correlations with the novel biomarker panel. These findings suggest that the investigated circulating biomarkers may reflect distinct and complementary biological pathways not fully captured by conventional risk markers, thereby offering additional mechanistic insight into coronary artery disease pathophysiology (Table 3).

### 2.4. Subgroup Analysis by Clinical Characteristics

Analysis of biomarker levels stratified by key clinical variables revealed no significant differences based on gender, smoking history, family history of coronary disease, or hypertension status within the coronary disease population (*p* > 0.05 for all comparisons).

Syntenin-1 levels showed no significant variation between male and female patients (20.7 ± 14.5 vs. 19.1 ± 9.2 ng/mL, *p* = 0.675), nor between smokers and non-smokers (20.3 ± 15.6 vs. 20.1 ± 10.6 ng/mL, *p* = 0.953). Similarly, the Phoenixin-14 concentrations remained stable across demographic subgroups, with median values showing minimal variation by gender [18.6 (14.6–24.7) vs. 18.6 (15.8–28.0) pg/mL for females and males, respectively; *p* = 0.715] and smoking status [18.3 (15.8–22.8) vs. 19.1 (14.6–28.3) pg/mL for smokers and non-smokers; *p* = 0.602] (Table 4).

### 2.5. Diagnostic Performance and Roc Analysis

ROC curve analysis revealed exceptional diagnostic performance for Phoenixin-14 in discriminating both severe and non-critical coronary artery disease from healthy controls. For severe coronary stenosis detection, Phoenixin-14 achieved an area under the curve of 0.969 (95% CI: 0.888–0.997, *p* < 0.001), demonstrating high diagnostic accuracy within this cohort; however, this level of performance may be influenced by sample characteristics and should be interpreted with caution. At the optimal cut-off value of ≤20.2 pg/mL, Phoenixin-14 demonstrated 86.7% sensitivity and 96.7% specificity, with positive and negative predictive values of 96.3% and 87.9%, respectively.

The diagnostic performance of Phoenixin-14 for non-critical coronary disease remained robust, with an AUC of 0.832 (95% CI: 0.713–0.916, *p* < 0.001). Internal validation by bootstrap resampling (2000 replications) indicated negligible optimism: the optimism-corrected AUC for Phoenixin-14 in discriminating severe stenosis from the controls was 0.970 (apparent AUC, 0.969; bootstrap 95% CI, 0.925–0.996) and 0.831 for non-critical disease (apparent AUC, 0.832; bootstrap 95% CI, 0.725–0.921). Using a cut-off threshold of ≤34.2 pg/mL, the biomarker achieved 93.3% sensitivity and 63.3% specificity for detecting non-critical CAD, with a positive predictive value (PPV) and negative predictive value (NPV) of 71.8% and 90.5%, respectively.

Syntenin-1 demonstrated moderate diagnostic utility for severe coronary stenosis (AUC 0.795, 95% CI: 0.668–0.890, *p* < 0.001), though with counterintuitive threshold relationships. At a cut-off value of ≤21.5 ng/mL, Syntenin-1 achieved 79.3% sensitivity and 82.8% specificity for severe CAD detection. For non-critical coronary disease, Syntenin-1 maintained modest discriminatory ability (AUC 0.720, 95% CI: 0.587–0.830, *p* = 0.002) with balanced sensitivity and specificity values of 72.4% and 75.9% at the optimal threshold of ≤25.2 ng/mL.

Alamandine showed limited but statistically significant diagnostic performance for severe coronary stenosis (AUC 0.661, 95% CI: 0.523–0.781, *p* = 0.029), achieving 100% sensitivity but only 28.6% specificity at the optimal cut-off of ≤119 pg/mL.

**Figure 2:**
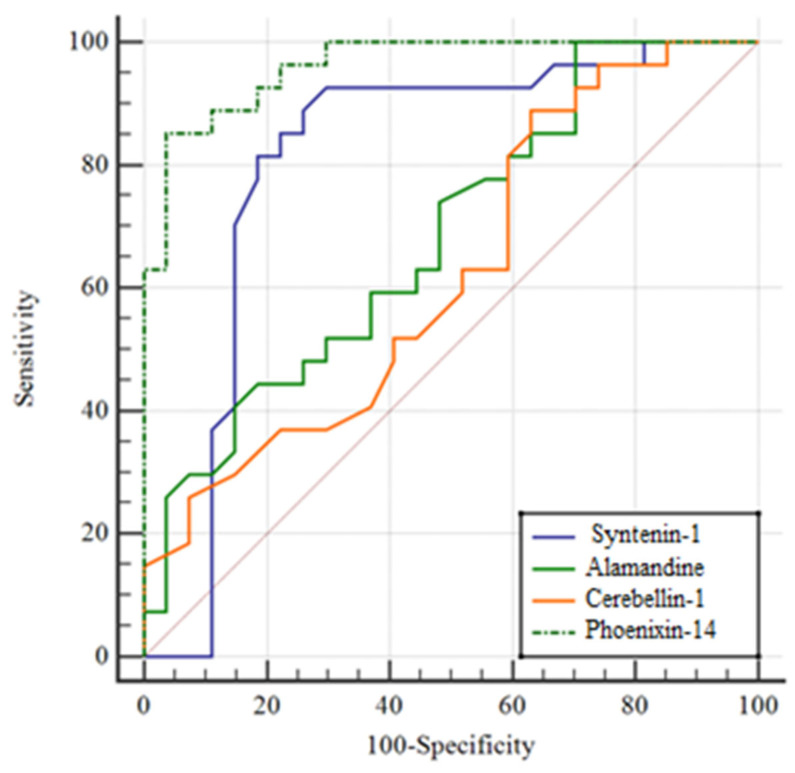
ROC curve of the four parameters for severe critical coronary stenosis.

**Figure 3:**
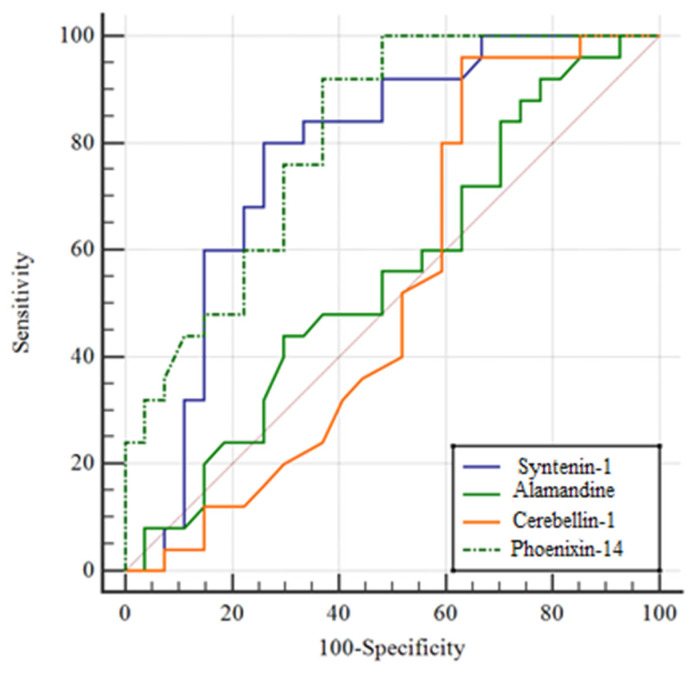
ROC curve of the four parameters for non-critical coronary stenosis.

Cerebellin-1 demonstrated poor diagnostic discrimination across all comparisons, with AUC values not significantly different from chance (severe CAD: AUC 0.597, *p* = 0.205; non-critical CAD: AUC 0.534, *p* = 0.670), confirming limited utility for coronary disease assessment (Table 5, Figure 2 and Figure 3).

In multivariable logistic regression, lower Phoenixin-14 was independently associated with the presence of CAD after adjustment for age and hypertension (adjusted OR per 1-SD increase, 0.077; 95% CI, 0.024–0.243; *p* < 0.001). Among patients with angiographically confirmed CAD, lower Phoenixin-14 was also independently associated with severe versus non-critical stenosis (adjusted OR per 1-SD increase, 0.036; 95% CI, 0.006–0.214; *p* < 0.001). Neither age nor hypertension was an independent predictor of disease severity (Table 6).

## 3. Discussion

A key finding of this study is the consistent, stepwise reduction in circulating Phoenixin-14 levels across the spectrum from angiographically normal coronary arteries to non-critical and severe CAD. This graded pattern supports the hypothesis that reduced Phoenixin-14 reflects increasing atherosclerotic burden rather than simple disease presence. Experimental evidence suggests that Phoenixin-14 may exert vasculoprotective effects through anti-inflammatory pathways, attenuation of oxidative stress, and nitric oxide-mediated endothelial regulation; however, clinical data in human coronary populations remain scarce [18,19,20,21,22]. In this context, the magnitude of diagnostic performance observed here is notable and compares favorably with established biomarkers that primarily reflect myocardial injury rather than chronic coronary atherosclerotic burden [3,5].

In contrast, Syntenin-1 exhibited an inverse and somewhat unexpected association with coronary artery disease, with significantly higher circulating concentrations observed in healthy controls compared with patients with both non-critical and severe CAD. Although Syntenin-1 is a multifunctional adaptor protein involved in intracellular trafficking, exosome biogenesis, and cell–cell communication, and has been implicated in inflammatory signaling cascades and endothelial regulation in experimental models, its role in human cardiovascular pathology remains incompletely characterized and largely speculative [23]. The observed reduction in circulating Syntenin-1 in CAD patients may reflect disease-related alterations in endothelial integrity, immune activation, or extracellular vesicle dynamics, all of which are known to be disrupted in atherosclerotic processes [24]. In particular, Syntenin-1 plays a key role in exosome formation through its interactions with syndecans and the endosomal sorting complex, suggesting that changes in its circulating levels could mirror broader dysregulation of intercellular communication networks in vascular disease. From a pathophysiological perspective, the directionality of this association is not yet clearly understood. An intriguing finding of the present study was the significantly lower circulating Syntenin-1 concentrations observed in patients with CAD compared with the controls. Although Syntenin-1 is known to play important roles in exosome biogenesis, intracellular signaling, cell adhesion, inflammatory regulation, and vascular homeostasis, its precise role in human coronary artery disease remains poorly understood. One possible explanation is that reduced circulating Syntenin-1 levels may reflect impaired exosome-mediated protective signaling, altered intercellular communication, endothelial dysfunction, or disease-related disturbances in vascular homeostasis. Alternatively, decreased circulating concentrations may result from increased utilization or sequestration of Syntenin-1 within activated vascular and immune tissues during atherosclerotic progression. It is also possible that the observed pattern reflects compensatory or context-dependent regulatory mechanisms that vary according to disease stage, inflammatory burden, or therapeutic interventions. Given the limited and occasionally conflicting evidence regarding circulating Syntenin-1 in cardiovascular disease, these interpretations remain speculative and should be interpreted with caution. Further mechanistic and prospective studies are required to clarify the biological significance of reduced Syntenin-1 levels in CAD and to determine whether this molecule plays a protective, adaptive, or disease-associated role in atherosclerosis [10].

Given these uncertainties, the biological plausibility of decreased Syntenin-1 in CAD warrants further mechanistic investigation. Future studies integrating cellular, translational, and clinical approaches are needed to clarify its role in endothelial dysfunction, inflammatory modulation, and extracellular vesicle-mediated signaling, and to determine whether Syntenin-1 represents a marker of vascular injury, a compensatory protective factor, or a bystander of systemic disease process.

Alamandine demonstrated a non-significant but consistent trend toward lower circulating concentrations in patients with coronary artery disease compared with the controls, in line with its proposed function as a component of the counter-regulatory, protective arm of the renin–angiotensin system. Mechanistically, Alamandine exerts its biological effects primarily through the MrgD, activating signaling pathways that promote vasodilation, inhibit vascular smooth muscle proliferation, and attenuate inflammatory and fibrotic responses within the cardiovascular system [25,26,27]. The modest and statistically limited discriminatory performance observed in the present study may be interpreted in several, not mutually exclusive, contexts.

First, pharmacological modulation represents a major potential confounder in real-world cardiovascular populations. Widespread use of renin–angiotensin system inhibitors, including angiotensin-converting enzyme inhibitors, angiotensin receptor blockers, and related therapies, may indirectly influence the endogenous balance of angiotensin peptides and their downstream metabolites, thereby blunting between-group differences in circulating Alamandine levels. Such medication-related effects may partially obscure the true biological gradient associated with disease severity [28]. Second, Alamandine may be more dynamically involved in acute vascular stress responses rather than chronic, stable atherosclerotic processes. While experimental evidence supports its vasoprotective and anti-inflammatory actions, its circulating levels in stable CAD populations may not adequately reflect local tissue-level activity or receptor-mediated signaling within the vascular wall. This dissociation between systemic concentrations and tissue-specific effects may limit its utility as a standalone biomarker for chronic disease burden [29]. Third, interindividual variability in renin–angiotensin system activity, metabolic status, and endothelial function may further contribute to overlapping concentration ranges across study groups. This biological heterogeneity, combined with the relatively small sample size, likely reduces the statistical power to detect subtle but potentially meaningful differences [30].

Taken together, these considerations suggest that Alamandine may be more reflective of dynamic regulatory activity within the alternative renin–angiotensin system rather than serving as a stable circulating marker of chronic coronary atherosclerosis. As a component of the non-classical RAS axis, Alamandine levels may be influenced, directly or indirectly, by pharmacological agents that modulate angiotensin processing, receptor interactions, or downstream enzymatic pathways. In particular, RAS inhibitors could potentially shift the equilibrium between classical and counter-regulatory RAS pathways, thereby affecting Alamandine concentrations through complex and not yet fully elucidated mechanisms. However, current evidence regarding the magnitude and direction of such medication-related effects in humans remains limited and inconsistent.

In the present study, detailed medication data were collected and compared across groups, and no significant differences were observed in the use of RAS inhibitors. Nonetheless, given the observational design of the study, we acknowledge that residual confounding related to pharmacotherapy cannot be entirely excluded. Therefore, the possible impact of RAS-targeting treatments on Alamandine levels should be taken into account when interpreting our findings, and warrants further investigation in controlled experimental and prospective clinical studies.

Cerebellin-1 did not demonstrate significant differences across study groups and showed limited diagnostic utility for distinguishing coronary artery disease severity. Although Cerebellin-1 is a secreted neuropeptide originally characterized in the central nervous system and subsequently detected in peripheral tissues, its physiological role outside the nervous system remains incompletely understood. In particular, its involvement in cardiovascular biology has not been clearly established, and available experimental evidence linking Cerebellin-1 to endothelial function, inflammation, or atherogenesis is limited and largely indirect [31,32]. The absence of significant intergroup differences in the present study suggests that circulating Cerebellin-1 levels are not substantially influenced by the presence or severity of coronary atherosclerosis, at least within the studied population. This may indicate that Cerebellin-1 does not participate meaningfully in the core pathophysiological pathways driving coronary artery disease, or that its potential cardiovascular effects are confined to tissue-specific or context-dependent mechanisms not reflected in systemic circulation.

Collectively, these findings highlight the heterogeneous behavior of emerging circulating biomarkers and underscore the complexity of CAD pathobiology. The superior performance of Phoenixin-14, in particular, supports the concept that neuropeptide- and peptide-based signaling pathways may provide novel and complementary insights beyond traditional risk factors and conventional biochemical markers. These observations also reinforce the potential value of multi-biomarker strategies that integrate distinct but biologically interconnected pathways for improved disease characterization. Importantly, the exceptionally high AUC observed for Phoenixin-14 should be interpreted in the context of methodological constraints, including limited sample size, potential spectrum bias, and lack of external validation. These factors may contribute to inflated estimates of diagnostic performance and underscore the need for cautious interpretation.

An additional notable finding was the lower body mass index observed in patients with severe CAD compared with those with non-critical disease, a pattern that may reflect the so-called “obesity paradox” described in cardiovascular populations. Alternatively, this may be influenced by residual confounding, differences in body composition, or selection bias inherent to angiography-based cohorts. Similarly, the absence of significant intergroup differences in lipid parameters may be related to background lipid-lowering therapy or the preselection of high-risk individuals, and does not diminish the established causal role of dyslipidemia in coronary atherosclerosis.

Several limitations warrant consideration. First, the single-center, cross-sectional design and relatively small sample size limit both statistical power and external validity. Second, the lack of multivariable adjustment precludes determination of whether Phoenixin-14 independently predicts CAD severity beyond established cardiovascular risk factors. Third, potential pharmacological confounding due to unrecorded or unadjusted medication effects (e.g., ACE inhibitors, antiplatelet agents) may have influenced the circulating biomarker levels. Fourth, coronary lesion severity was defined solely by angiographic stenosis rather than physiological indices such as fractional flow reserve, which may introduce the misclassification of lesion significance. Finally, the absence of longitudinal follow-up prevents the assessment of prognostic utility and temporal dynamics of these biomarkers. Future research should prioritize large-scale, multicenter prospective studies with standardized sampling protocols, comprehensive adjustment for confounders, and integration of functional coronary assessment. Mechanistic studies are also required to elucidate the biological role of Phoenixin-14 and related peptides in atherosclerosis. Although bootstrap-based internal validation suggested limited optimism, the discriminatory performance and proposed cut-off values were derived from a relatively small, single-center cohort and should therefore be interpreted with caution pending external prospective validation. In addition, the weak associations observed between Phoenixin-14 and individual clinical parameters lost statistical significance after correction for multiple testing, indicating limited clinical relevance.

Within the present study population, Phoenixin-14 levels were associated with CAD severity and showed encouraging diagnostic discrimination. Nevertheless, the absence of multivariable analyses precluded an assessment of whether these associations were independent of potential confounding factors. Consequently, the findings should be considered hypothesis-generating rather than definitive. Future large-scale, multicenter prospective studies with comprehensive adjustment for confounders are needed to validate these observations and determine the clinical utility of Phoenixin-14 as a diagnostic biomarker for CAD.

## 4. Materials and Methods

Patients were consecutively screened and enrolled from individuals presenting to the Cardiology Outpatient Clinic of Fethi Sekin City Hospital who underwent coronary angiography for diagnostic or therapeutic purposes between April 2025 and the end of the recruitment period. Written informed consent was obtained from all participants prior to enrollment. Because of the exploratory nature of the investigation, sample size determination was based on feasibility and consecutive patient recruitment during the predefined study period rather than formal a priori power analysis. Nevertheless, the final cohort size was comparable to those reported in previous preliminary biomarker studies and was considered sufficient for an estimation of preliminary effect sizes to guide future adequately powered investigations.

Participants were stratified according to coronary angiographic findings into three groups, each comprising 30 individuals. The critical stenosis group included patients with stable angina pectoris and ≥70% luminal stenosis in at least one major epicardial coronary artery. The non-critical stenosis group consisted of patients with <70% coronary stenosis, whereas the control group comprised individuals with angiographically normal coronary arteries and no evidence of coronary artery disease. Stable angina pectoris was defined according to contemporary guideline-based criteria as exertional chest discomfort relieved by rest and/or nitrates in the absence of features suggestive of acute coronary syndrome. Indications for coronary angiography included typical anginal symptoms, abnormal noninvasive stress testing, or high clinical suspicion of obstructive coronary artery disease. Patients with acute coronary syndrome, diabetes mellitus, morbid obesity, statin users, previous coronary artery disease, prior coronary revascularization, heart failure, cardiomyopathy, or history of early menopause were excluded from the study.

Venous blood samples (5 mL) were obtained from all participants within 24 h of hospital admission following overnight fasting and were collected according to standardized venipuncture and serum preparation procedures routinely used in the institutional biochemistry laboratory [33]. Although fasting conditions were standardized, the exact timing of blood collection was not uniform; therefore, potential circadian variation in biomarker concentrations cannot be completely excluded. Blood samples were centrifuged at 4000 rpm for 5 min, and the separated serum aliquots were immediately stored at −20 °C until biochemical analysis.

Routine biochemical and hematological parameters, including lipid profiles, were analyzed using standardized automated analyzers in the central laboratory, which operates under regular internal and external quality-control procedures. Serum concentrations of Phoenixin-14, Alamandine, Cerebellin-1, and Syntenin-1 were quantified using commercially available enzyme-linked immunosorbent assay (ELISA) kits according to the manufacturers’ protocols. Demographic and clinical characteristics were systematically recorded for all participants, including age, sex, body weight, height, body mass index, systolic and diastolic blood pressure, smoking status, family history of coronary artery disease, hypertension, and hyperlipidemia. Hematological and biochemical measurements obtained within 24 h of admission were subsequently compared among the study groups. Statistical analyses were performed to evaluate intergroup differences and determine the significance of observed associations.

### 4.1. Preparation and Storage of Biological Samples

Following an overnight fast, venous blood samples (5 mL) were collected from all participants using standardized venipuncture procedures into biochemistry tubes containing aprotinin to minimize proteolytic degradation of circulating peptides. Blood samples were centrifuged at 4000 rpm (1792× *g*) for 5 min, and the separated serum fractions were aliquoted into sterile Eppendorf tubes. Serum samples were subsequently stored at −20 °C until biochemical analyses were performed. Before analysis, frozen samples were thawed at +4 °C and equilibrated to room temperature (23–25 °C) under controlled laboratory conditions.

### 4.2. Measurement of Circulating Biomarkers and Elisa Assays

Serum concentrations of Phoenixin-14, Alamandine, Cerebellin-1, and Syntenin-1 were quantified using commercially available ELISA kits in accordance with the manufacturers’ instructions. Phoenixin-14 levels were measured using the Human Phoenixin-14 ELISA Kit (Sunlong Biotech Co., Ltd., Shanghai, China; Catalog No: QSD4167Hu), with a reported sensitivity of 1 pg/mL and an assay range of 10–400 pg/mL. Alamandine concentrations were determined using the Human Alamandine ELISA Kit (Sunlong Biotech Co., Ltd., Shanghai, China; Catalog No: QSD4070Hu), with a sensitivity of 1 ng/L (0.001 ng/mL) and a detection range of 0.004–0.200 ng/mL. Cerebellin-1 concentrations were analyzed using the Human Cerebellin-1 ELISA Kit (Shanghai Sunred Biological Technology, Shanghai, China; Catalog No: 201-12-3438), with a sensitivity of 1 pg/mL and a detection range of 5–1500 pg/mL. Syntenin-1 levels were assessed using the Human Syntenin-1 ELISA Kit (Shanghai Sunredbio Technology Co., Ltd., Shanghai, China; Catalog No: 201-12-5043), with a sensitivity of 0.479 ng/mL and an assay range of 0.5–150 ng/mL.

Calibration curves were generated for each assay using manufacturer-provided standards, and internal quality-control samples were included in every analytical run. All measurements were performed in duplicate, and laboratory personnel were blinded to the participants’ clinical and angiographic data. The intra-assay and inter-assay coefficients of variation were <10% and <12%, respectively, confirming acceptable analytical reproducibility and precision.

### 4.3. Analysis of Phoenixin-14, Alamandine, Cerebellin-1, and Syntenin-1

Quantitative analyses of Phoenixin-14, Alamandine, Cerebellin-1, and Syntenin-1 were performed using ELISA methodology in accordance with both the manufacturers’ protocols and previously published methodological recommendations [33]. Automated washing procedures were carried out using a Bio-Tek ELX50 microplate washer (BioTek Instruments, Winooski, VT, USA). Optical density measurements were obtained at 450 nm using a ChroMate microplate reader (Awareness Technology Inc., Palm City, FL, USA), and analyte concentrations were calculated from standard calibration curves.

Potential confounding variables, including concomitant pharmacological therapies such as statins, angiotensin-converting enzyme inhibitors, and antiplatelet agents, were not systematically adjusted for during statistical analyses. Although major demographic and clinical variables were documented, the potential influence of these factors on circulating biomarker concentrations cannot be entirely excluded.

### 4.4. Statistical Analysis

All statistical analyses were performed using IBM SPSS Statistics for Windows, version 22.0 (IBM Corp., Armonk, NY, USA). Categorical variables are presented as frequencies and percentages, whereas continuous variables are expressed as mean ± standard deviation (SD) for normally distributed data or median with interquartile range (IQR; 25th–75th percentiles) for non-normally distributed variables. The distributional properties of continuous variables were assessed using the Kolmogorov–Smirnov test in combination with a visual inspection of histograms and probability plots. Homogeneity of variance was evaluated prior to parametric testing.

Comparisons between categorical variables were performed using Pearson’s chi-square test or Fisher’s exact test, as appropriate. For comparisons of continuous variables between two independent groups, the Student’s t-test was used for normally distributed data, whereas the Mann–Whitney U test was applied for non-parametric variables. For analyses involving more than two groups, one-way analysis of variance (ANOVA) was employed for parametric variables and the Kruskal–Wallis test for non-parametric variables. Correlations between circulating biomarker concentrations and continuous clinical variables were assessed using Spearman rank correlation analysis because several biomarker parameters demonstrated non-normal distribution characteristics.

ROC curve analysis was performed to evaluate the diagnostic performance of Phoenixin-14, Alamandine, Cerebellin-1, and Syntenin-1 in discriminating coronary artery disease phenotypes. AUC with corresponding 95% confidence intervals (CIs) was calculated for each biomarker. The internal validity of the ROC-derived discrimination was assessed by bootstrap resampling (2000 replications) to estimate optimism-corrected AUC values. Optimal threshold values were identified using Youden’s index to maximize combined sensitivity and specificity. Diagnostic performance indices, including sensitivity, specificity, PPV, and NPV were subsequently calculated for clinically relevant cut-off values. Multivariable binary logistic regression was used to assess whether Phoenixin-14 was independently associated with the presence of CAD (any CAD vs. controls), and among patients with CAD, with disease severity (severe vs. non-critical stenosis). Models were adjusted for age and hypertension and were deliberately kept parsimonious to maintain an events-per-variable ratio of at least 10 and to limit overfitting; results are reported as adjusted odds ratios (aOR) with 95% confidence intervals (CIs) per 1-standard-deviation change in Phoenixin-14. To control the family-wise type I error across the four candidate biomarkers, omnibus group comparisons were additionally adjusted using the Bonferroni–Holm procedure, and post hoc pairwise comparisons were performed using Dunn’s test with Bonferroni adjustment. Correlation *p*-values were corrected for multiple testing across all pairwise comparisons using the Benjamini–Hochberg false discovery rate (FDR) procedure. All statistical tests were two-tailed, and a *p*-value < 0.05 was considered statistically significant.

## 5. Conclusions

In this exploratory, single-center study, we evaluated four circulating biomarkers—Phoenixin-14, Alamandine, Syntenin-1, and Cerebellin-1—for their potential utility in the diagnosis and severity stratification of coronary artery disease (CAD). Among these candidates, Phoenixin-14 demonstrated the strongest and most consistent performance, exhibiting excellent discriminatory ability for CAD severity (AUC: 0.969) and a robust inverse association with angiographically defined disease burden. Although bootstrap-based internal validation suggested limited optimism and reduced the likelihood of major overfitting, these findings remain preliminary due to the modest sample size and absence of external validation. Accordingly, the reported diagnostic accuracy and proposed thresholds should be interpreted cautiously and require confirmation in independent, multicenter prospective cohorts before clinical translation can be considered. Syntenin-1 showed a moderate inverse association with CAD, suggesting a potentially important but incompletely understood role in vascular and intercellular communication pathways. Alamandine demonstrated modest discriminatory performance, which may reflect modulation by disease stage and/or pharmacological influences within the renin–angiotensin system, although these mechanisms remain speculative. In contrast, Cerebellin-1 did not show clinically relevant discriminatory value in this cohort.

Overall, our findings support the concept that multi-biomarker strategies may improve the phenotypic characterization of CAD beyond conventional clinical assessment. Among the investigated markers, Phoenixin-14 emerges as the most promising candidate for further translational investigation, particularly in larger, well-designed studies incorporating external validation and clinical outcome-based endpoints. Until such evidence is available, its clinical utility should be considered investigational.

## Figures and Tables

**Table 1:** Baseline clinical characteristics and laboratory parameters by study group.

Characteristic	Severe CAD (*n* = 30)	Non-Critical CAD (*n* = 30)	Healthy Controls (*n* = 30)	*p*-Value
**Demographics**				
Male gender, n (%)	20 (66.7)	21 (70.0)	16 (53.3)	0.366
Age, years	62.6 ± 11.6	61.6 ± 10.8	59.6 ± 9.7	0.562
Height, cm (min–max)	171.0 (160.0–177.0)	167.5 (158.0–172.0)	163.0 (158.0–170.0)	0.095
Weight, kg	74.7 ± 11.9	81.7 ± 10.8	74.9 ± 12.7	0.037
Body mass index, kg/m^2^	26.0 ± 3.4	29.6 ± 3.9	27.8 ± 4.5	0.003
**Cardiovascular Risk Factors**				
Current smoking, *n* (%)	14 (46.7)	12 (40.0)	10 (33.3)	0.574
Family history of CAD, *n* (%)	20 (66.7)	17 (56.7)	19 (63.3)	0.718
Hypertension, *n* (%)	18 (60.0)	21 (70.0)	10 (33.3)	0.013
**Hemodynamic Parameters**				
Systolic BP, mmHg	136.3 ± 18.3	134.1 ± 14.4	122.1 ± 16.6	0.003
Diastolic BP, mmHg	78.9 ± 12.9	81.4 ± 10.6	74.1 ± 10.4	0.047
**Laboratory Parameters**				
Glucose, mg/dL (min–max)	105.0 (92.0–140.0)	115.5 (91.0–140.0)	108.0 (102.0–137.0)	0.614
Hemoglobin, g/dL	14.0 ± 1.6	14.8 ± 1.3	13.9 ± 1.4	0.065
Creatinine, mg/dL (min–max)	0.8 (0.7–1.0)	0.8 (0.6–1.0)	0.7 (0.6–0.7)	0.001
eGFR, mL/min/1.73 m^2^	83.2 ± 21	85.2 ± 12.9	99.2 ± 12.3	<0.001
Sodium, mEq/L (min–max)	139.0 (137.0–140.0)	138.5 (137.0–142.0)	139.0 (137.0–140.0)	0.853
Potassium, mEq/L (min–max)	4.4 (4.2–4.7)	4.3 (4.1–4.5)	4.2 (3.9–4.3)	0.064
Uric acid, mg/dL (min–max)	5.1 (4.6–6.4)	5.1 (4.2–6.0)	4.7 (3.7–5.3)	0.048
Albumin, g/dL (min–max)	42.0 (39.0–44.0)	42.5 (41.0–44.0)	43.0 (42.0–44.0)	0.313
C-reactive protein, mg/L (min–max)	2.8 (1.1–5.3)	3.0 (1.1–5.3)	2.3 (1.0–5.1)	0.779
**Lipid Profile**				
LDL cholesterol, mg/dL	103.6 ± 37.2	116.6 ± 40.5	118.7 ± 33.5	0.240
HDL cholesterol, mg/dL	39.4 ± 8.2	45.4 ± 9.1	49.2 ± 10.0	<0.001
Triglycerides, mg/dL	144.0 (115.0–220.0)	140.0 (100.0–196.0)	149.0 (94.0–200.0)	0.853
Total cholesterol, mg/dL	177.0 ± 45	192.6 ± 46.6	199.0 ± 39.7	0.141

BP: blood pressure, CAD: coronary artery disease, eGFR: estimated glomerular filtration rate, HDL: high-density lipoprotein, LDL: low-density lipoprotein.

**Table 2:** Medication use across study groups.

Medication Class	Severe CAD (*n* = 30)	Non-Critical CAD (*n* = 30)	Healthy Controls (*n* = 30)	*p*-Value
ACE inhibitor	3 (10.0)	4 (13.3)	3 (10.0)	1.000
Angiotensin receptor blocker	8 (26.7)	9 (30.0)	7 (23.3)	0.951
Beta-blocker	3 (10.0)	4 (13.3)	0 (0.0)	0.206
Calcium channel blocker	4 (13.3)	4 (13.3)	0 (0.0)	0.120

Data are *n* (%). *p*-values from Fisher’s exact test (Freeman–Halton extension). Medication use did not differ significantly between groups for any class, and was balanced between the severe and non-critical CAD subgroups. CAD: coronary artery disease.

**Table 3:** Correlation matrix of circulating biomarkers and clinical parameters in patients with coronary artery disease (*n* = 60).

Parameter	Syntenin-1	Alamandine	Cerebellin-1	Phoenixin-14
	*r* (*p* Value)	*r* (*p* Value)	*r* (*p* Value)	*r* (*p* Value)
**Biomarker Correlations**				
Alamandine	−0.111 (0.420)	—	—	—
Cerebellin-1	0.207 (0.130)	−0.001 (0.994)	—	—
Phoenixin-14	−0.044 (0.741)	0.725 (<0.001)	0.114 (0.398)	—
**Anthropometric Parameters**				
Age, years	0.110 (0.411)	0.039 (0.780)	−0.061 (0.654)	−0.166 (0.205)
Height, cm	−0.096 (0.474)	−0.077 (0.575)	−0.048 (0.721)	−0.040 (0.761)
Weight, kg	0.097 (0.468)	0.173 (0.205)	0.040 (0.768)	0.361 (0.005)
Body mass index, kg/m^2^	0.130 (0.329)	0.209 (0.125)	0.060 (0.657)	0.382 (0.003)
**Hemodynamic Parameters**				
Systolic BP, mmHg	0.049 (0.715)	−0.113 (0.410)	0.216 (0.107)	−0.103 (0.436)
Diastolic BP, mmHg	0.076 (0.568)	−0.062 (0.651)	0.133 (0.324)	−0.060 (0.648)
**Laboratory Parameters**				
Glucose, mg/dL	−0.006 (0.967)	0.308 (0.022)	−0.080 (0.556)	0.176 (0.179)
Hemoglobin, g/dL	0.021 (0.876)	−0.076 (0.583)	0.010 (0.943)	0.182 (0.163)
Creatinine, mg/dL	−0.099 (0.458)	−0.127 (0.355)	0.111 (0.412)	−0.143 (0.274)
eGFR, mL/min/1.73 m^2^	0.014 (0.919)	−0.024 (0.862)	−0.177 (0.188)	0.040 (0.763)
Sodium, mEq/L	−0.114 (0.394)	−0.201 (0.141)	0.128 (0.342)	−0.016 (0.903)
Potassium, mEq/L	0.076 (0.572)	0.098 (0.475)	−0.015 (0.914)	−0.165 (0.209)
Uric acid, mg/dL	0.041 (0.758)	−0.054 (0.695)	−0.074 (0.584)	−0.057 (0.667)
Albumin, g/dL	−0.107 (0.422)	0.033 (0.813)	0.203 (0.130)	0.158 (0.227)
C-reactive protein, mg/L	−0.070 (0.599)	0.003 (0.981)	0.025 (0.856)	0.079 (0.548)
**Lipid Profile**				
LDL cholesterol, mg/dL	0.210 (0.114)	−0.090 (0.512)	0.077 (0.571)	0.081 (0.539)
HDL cholesterol, mg/dL	0.056 (0.677)	0.088 (0.524)	0.133 (0.323)	0.101 (0.444)
Triglycerides, mg/dL	0.093 (0.487)	−0.261 (0.054)	0.262 (0.049)	−0.151 (0.249)
Total cholesterol, mg/dL	0.250 (0.059)	−0.147 (0.285)	0.180 (0.180)	0.045 (0.734)

Statistical analysis: Spearman correlation coefficients. BP: blood pressure, eGFR: estimated glomerular filtration rate, HDL: high-density lipoprotein, LDL: low-density lipoprotein. Only the Phoenixin-14–Alamandine correlation remained significant after Benjamini–Hochberg FDR correction; all other associations were non-significant after adjustment for multiple comparisons.

**Table 4:** Comparison of molecule levels according to sex, smoking, family history, and hypertension in the coronary stenosis group.

Parameter		Syntenin-1		Alamandine		Cerebellin-1		Phoenixin-14	
		Mean ± SD	*p* *	Median (IQR)	*p* **	Median (IQR)	*p* **	Median (IQR)	*p* **
**Sex**	Female	19.1 ± 9.2	0.675	69.0 (52.8–95.8)	0.730	1.6 (1.1–2.8)	0.986	18.6 (14.6–24.7)	0.715
	Male	20.7 ± 14.5		63.7 (48.4–100.7)		1.7 (0.8–2.4)		18.6 (15.8–28.0)	
**Smoking**	Yes	20.3 ± 15.6	0.953	60.7 (47.0–86.1)	0.131	1.6 (0.8–2.0)	0.357	18.3 (15.8–22.8)	0.602
	No	20.1 ± 10.6		75.0 (52.8–111.6)		1.9 (1.1–2.8)		19.1 (14.6–28.3)	
**Family history**	Yes	19.3 ± 13.5	0.515	61.6 (49.5–88.9)	0.302	1.6 (1.0–2.8)	0.902	18.4 (14.1–21.0)	0.176
	No	21.6 ± 12.1		74.6 (48.4–118.8)		1.8 (0.9–2.3)		21.3 (17.0–29.9)	
**Hyper-tension**	Yes	21.0 ± 14.5	0.499	75.0 (48.1–97.7)	0.997	1.7 (1.1–2.7)	0.798	18.5 (14.1–25.9)	0.420
	No	18.6 ± 9.4		60.9 (49.1–99.5)		1.9 (0.8–2.3)		20.2 (17.1–28.0)	

* Student’s *t*-test and ** Mann–Whitney U test were applied.

**Table 5:** Specificity and sensitivity of measured parameters in identifying coronary stenosis.

Variable	AUC	*p*	95% Confidence Interval		Sensitivity (%)	Specificity (%)	PPV (%)	NPV (%)
			Lower Limit	Upper Limit				
Diagnosis of significant coronary stenosis *								
Syntenin-1 ≤ 21.5	0.795	<0.001	0.668	0.890	79.3	82.8	82.1	80.0
Alamandine ≤ 119	0.661	0.029	0.523	0.781	100.0	28.6	59.2	100.0
Cerebellin-1 ≤ 2.9	0.597	0.205	0.458	0.725	85.7	37.9	57.1	73.3
Phoenixin-14 ≤ 20.2	0.969	<0.001	0.888	0.997	86.7	96.7	96.3	87.9
Diagnosis of non-critical coronary stenosis **								
Syntenin-1 ≤ 25.2	0.720	0.002	0.587	0.830	72.4	75.9	75.0	73.3
Alamandine ≤ 61.6	0.541	0.607	0.400	0.678	46.2	67.9	57.1	57.6
Cerebellin-1 > 2.9	0.534	0.670	0.398	0.666	17.2	62.1	31.2	42.9
Phoenixin-14 ≤ 34.2	0.832	<0.001	0.713	0.916	93.3	63.3	71.8	90.5

AUC: area under the ROC curve, PPV: positive predictive value, NPV: negative predictive value. * Significant coronary stenosis diagnosis ** Non-critical coronary stenosis diagnosis.

**Table 6:** Multivariable logistic regression analysis of Phoenixin-14 and clinical covariates.

Variable	aOR	95% CI	*p* Value
**Model 1—Presence of CAD (any CAD vs. controls); *n* = 90, 60 events**			
Phoenixin-14 (per 1-SD increase)	0.077	0.024–0.243	<0.001
Age (per year)	1.04	0.98–1.10	0.240
Hypertension (present vs. absent)	3.86	1.00–14.93	0.050
**Model 2—Disease severity (severe vs. non-critical stenosis); *n* = 60, 30 events**			
Phoenixin-14 (per 1-SD increase)	0.036	0.006–0.214	<0.001
Age (per year)	1.02	0.96–1.08	0.579
Hypertension (present vs. absent)	0.32	0.07–1.40	0.130

aOR: adjusted odds ratio; CI: confidence interval; CAD: coronary artery disease; SD: standard deviation. Odds ratios for Phoenixin-14 are expressed per 1-SD increase (SD = 14.3 pg/mL).

## Data Availability

The original contributions presented in this study are included in the article. Further inquiries can be directed to the corresponding author.

## Abbreviations

The following abbreviations are used in this manuscript:

ANOVA	One-Way Analysis of Variance
AUC	Area Under the ROC Curve
CAD	Coronary Artery Disease
CIs	Confidence Intervals
CVD	Cardiovascular Disease
ELISA	Enzyme-Linked Immunosorbent Assay
HDL	High-Density Lipoprotein
IQR	Interquartile Range
MrgD	Mas-related G protein-coupled receptor D
NPV	Negative Predictive Value
PPV	Positive Predictive Value
RAS	Renin–Angiotensin System
ROC	Receiver Operating Characteristic
SD	Standard Deviation
SMIM20	Small Integral Membrane Protein 20
